# Harnessing the Spatial Foundation of Mind in Breaking Vicious Cycles in Anxiety, Insomnia, and Depression: The Future of Virtual Reality Therapy Applications

**DOI:** 10.3389/fpsyt.2021.645289

**Published:** 2021-07-08

**Authors:** Ravinder Jerath, Connor Beveridge

**Affiliations:** Charitable Medical Healthcare Foundation, Augusta, GA, United States

**Keywords:** virtual reality therapy, spatial cognition, mind-body therapies, mind, vicious cycle, anxiety, depression, insomnia

## Abstract

Mental Illnesses, particularly anxiety, insomnia, and depression often involve vicious cycles which are self-perpetuating and can trap one into a more chronic state. For example in the case of insomnia, sympathetic overactivity, intrusive thoughts, and emotional instability due to sleep loss can perpetuate further sleep loss the next night and so on. In this article, we put forward a perspective on breaking these vicious cycles based on preeminent theories in global and spatial cognition, that the foundation of the conscious mind is a spatial coordinate system. Based on this we discuss the potential and future of virtual reality therapeutic applications which utilize massive virtual spaces along with biofeedback designed to help break perpetual cycles in depression, anxiety, and insomnia. “Massive spaces” are those which are truly expansive such as when looking to the clear night sky. These virtual realities may take the form of a night sky, fantastical cosmic scenes, or other scenes such as mountain tops. We also hope to inspire research into such a spatial foundation of mind, use of perceived massive spaces for therapy, and the integration of biofeedback into virtual therapies.

## Introduction

Virtual reality (VR) is a computer generated simulation that recreates or imitates a realistic environment via sensations delivered to a person via a headset and/or other devices (1). By constructively engaging in certain cognitive activities with VR, individuals may be able to redirect intrusive thoughts, engage in cognitive rehabilitation, and become immersed in virtual situations that prevent or provide therapeutic effect to mental illnesses. There are different types of VR therapies which have shown to have therapeutic effect for mental illnesses. The most studied and widely used is exposure or habituation therapy, which exposes users to the source of their disorder in a safe, virtual environment (1). This form of therapy has shown useful in disorders in which exposure and modification of the mental fear structure by reduction in association with harm and danger such as PTSD (2, 3), phobias (4, 5), and anxieties (6, 7). With exposure therapy, patients can reproduce the situation that brings them fear and face it, and with habitual exposure, the threshold of anxiety and fear increases (1). Interactive VR therapy puts users as active participants in the virtual world and has been shown to have therapeutic benefits on social skills in those with schizophrenia (8) and autism (6, 9) by putting them in important interactive social situations such as job interviews (10) and allowing them to practice expressing and reading appropriate social interactions (11). Immersive VR based cognitive training has shown very useful in improving cognitive faculties in those with dementia and other cognitive issues (12, 13). VR applications that allow users to have a virtual body and interact with friends, family, and others in a virtual world can also improve mental health and well-being in those who are socially isolated due to neurodegenerative diseases, brain injuries, or other conditions (14). In this article, we propose a novel form of VR therapy which if technological innovation permits, would provide users with an experience of spatially expansive environments.

## A Vicious Cycle

The psychiatric disorders of anxiety, depression, anger, and insomnia have an intimate relationship with chronic stress as well as with each other. People with major depression or anxiety are likely to suffer from insomnia and sleep deprivation can trigger or increase the severity of depression and anxiety (15), in part by impairing top-down inhibitory networks and thus giving leeway to unpleasant, intrusive thoughts (16). Throughout the literature, these conditions are described as often occurring within, or as a part of, a vicious cycle (16–27). The symptoms of these conditions, the most significant being distress, may perpetuate and compound the conditions further, often leading to such a vicious cycle of chronic illness which may further feed depression, anxiety, and insomnia, often simultaneously (28, 29). For instance, in the case of anger, guilt and fear of one's own reactions can direct anger inwards, lowering self-esteem, thus triggering defense mechanisms which further prevent appropriate expression of anger and lead to other emotional disorders (25). These cycles can thus result in these conditions becoming independent of their origin over time, with bodily activity playing a significant role in their maintenance.

A vicious cycle of depression may arise *via* multiple pathways. In the vulnerable it often arises via the reciprocal relationship between depressed mood and negative thinking which compounds and reinforces initial depression (20). The autoimmune troubles brought about as a result of the chronic stress of depression or singular but powerful depressive events can lead to inflammatory conditions which cause neurotoxic changes in the brain making it much more prone to major depression (30). The self-reinforcing cycle of impaired sleep, intrusive thoughts, and consequently emotional distress likely plays a major role in major depression via disrupted inhibitory control networks (16, 31). Rumination is a common characteristic observed in depression (and anxiety) in which one responds to depression with compulsive and repetitive over-analysis on the meaning, causes, and consequences of the depression (32). It further fuels depression by enhancing negative thinking, impairing daily thinking and instrumental behavior, and eroding social ties and supports (32). Rumination alone is enough to trap one in a cycle of depression and/or anxiety; however, one's high-level beliefs of the world and self may further feed such a vicious cycle (33).

People always carry high-level beliefs about themselves and the world which may be engrained in top-down cortical rhythms and hierarchies (34). These predictions, expectations, and beliefs are called “priors,” and they often tighten in response to significant stress and uncertainty (35). Everyday priors include perceptual beliefs formed from experience such as perceiving convex spheres as concave. Priors can become overweighted into maladaptive beliefs (36) such as that one may never find love, or in the case of addiction that one may not be able to get through the day without a cigarette. Such pervasive negative priors have long been considered a core feature of depression (33, 37). Stress and uncertainty caused by depressive moods and experiences can lead to the tightening of negative priors which overtime can become pervasive and develop a vicious cycle in which these priors trigger a deeper depression which stimulates the formulation of even more revision resistant priors. By relaxing abnormal and harmful priors, possibly by disrupting the brain networks that carry them, the cycle may be attenuated (38, 39).

**Figure 1:**
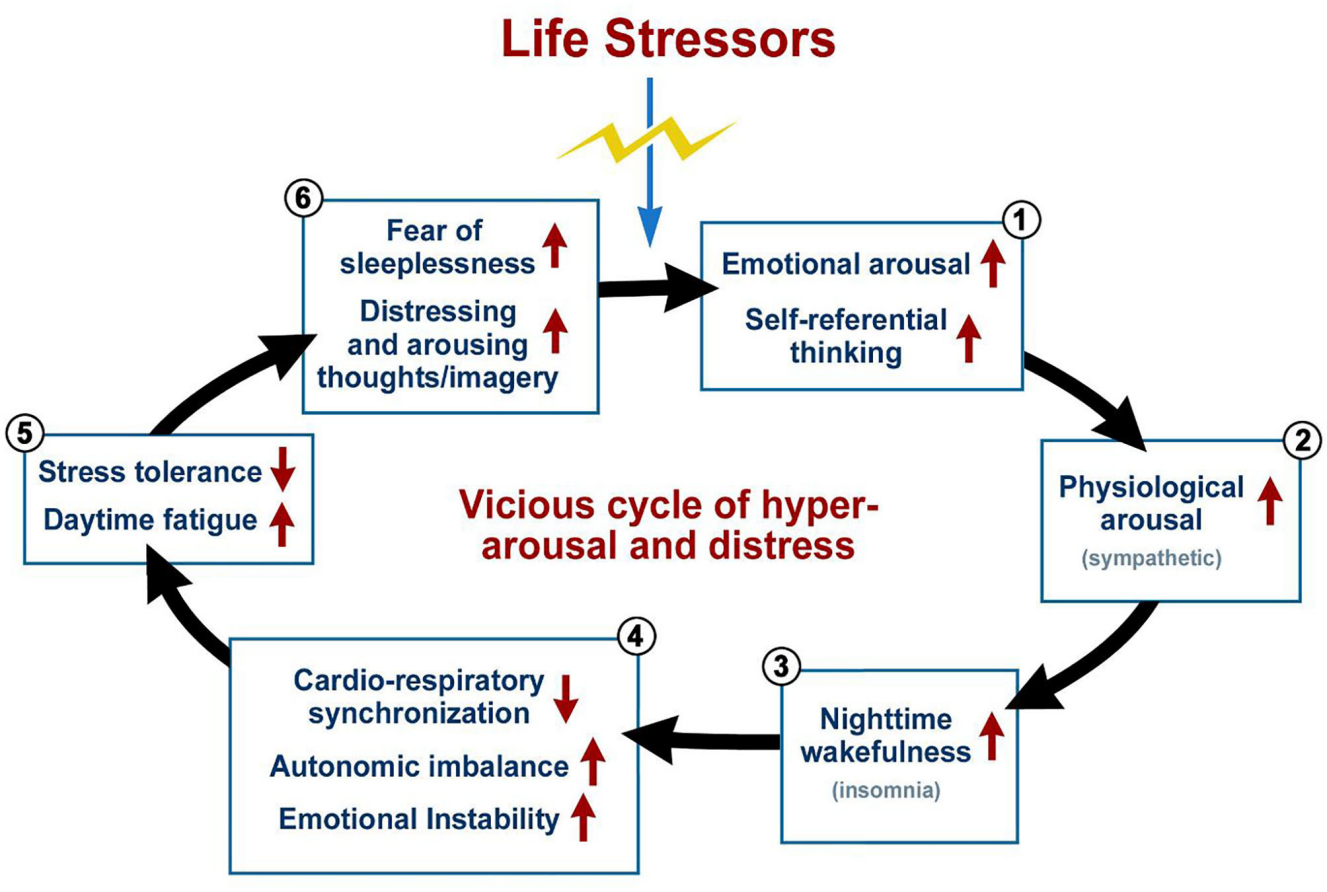
A vicious cycle in insomnia. This cyclic graph illustrates the vicious cycle in insomnia but is partially applicable to anxiety and depression as well, distress being a key feature of all three conditions. Such a cycle often begins with a significant life stressor which triggers intense emotional distress and even self-referential thinking which leads to arousal. The cycle begins after this event which causes initial insomnia via these reactions. Sleep deprivation leads to daytime bodily and emotional imbalances and instabilities which increase daytime fatigue and likelihood of becoming more distressed. Emotional dysregulation, impaired inhibitory control networks, and the hyper aroused state of mind can cause further distressing and intrusive thoughts as one tries to fall asleep which perpetuates a further round of insomnia. All of the negative results of sleep deprivation will likely cause the sufferer to develop a potentially severe fear of sleeplessness or they may experience significant pressure to fall asleep, all leading to a growing trouble falling asleep at night and thus perpetuating the cycle. Adapted from Jerath et al. (29).

States of the body and the resulting interoceptive perceptions greatly impact mental states, behavior, and emotions (40–42). The sympathetic branch of the autonomic nervous system is a major peripheral neural pathway activated by stress, and often in the conditions of insomnia, depression, and anxiety it can become continuously active with negligible counteraction by the parasympathetic branch (30, 43) leading to further sleep disturbances (44) and distress related conditions. The flight-or-fight response of sympathetic activity is evolutionarily purposed for short-term activation in preparation for immediate dangers, however, in modern times this activity can remain chronically elevated due to incessant background distress of modern life (45, 46). This sympathetic hypertonicity or hyperarousal can be thought of as an “evolutionary mismatch disease,” in which such autonomic traits were advantageous to life in the wild, but are mismatched and maladaptive to post agricultural and industrial revolution lifestyles which emerged rapidly compared to the several million years of humanoid evolution (47, 48). This hyperarousal plays an important bi-directional role in fueling vicious cycles of mental illness by promoting, and being promoted by them (22, 28, 30, 49, 50). Nearly all etiological models of insomnia include hyperarousal as a key aspect (26). Such a vicious cycle of insomnia is illustrated in Figure 1. Due to the impairment of top-down inhibitory control, sleep deprivation can impair negative memory suppression, allowing aversive thoughts to pervade the mind resulting in the development of anxiety (16). Fear of anxiety symptoms can develop often resulting in deepening anxiety. Certain individuals are more likely to selectively pay attention to these symptoms and this shift of attention inward fuels the cycle of anxiety even further (51). Once a cycle is started, negative cognitive responses such as self-focused attention, exaggerated attention toward potential threats (27), and overweighting of negative consequences can act to maintain the cycle (52). These cognitive responses produce exaggerated information which when processed, exacerbate information processing biases even further (51). Behavior becomes more inhibited and avoidant and reactivity increases, and overtime, all of these patterns become rigid (27). The relationship between sugar consumption and anxiety is also bi-directional with such consumption as a form of self-medication for anxiety leading to increased vulnerability to stress (53).

## The Therapeutic Capacity of Slow Deep Breathing, Mental Imagery, and Biofeedback

Slow, deep breathing techniques have been shown to be effective in improving symptoms of anxiety, depression (54–56), insomnia (57), and sympathetic hypertonicity (58). This has been demonstrated in magnetic resonance imaging studies (59). These conditions are very common in the modern world, but are strikingly prevalent in those with chronic breathing disorders, suggesting and intimate link between them (60). Mindful breathing exercises are associated with lower intensity of rumination, repetitive negative thinking, and depression (61). Breathing exercises improve heart-rate variability with lower variability associated with depression (57) and poor autonomic balance (62), while also promoting positive mood an relaxation (63, 64).

Positive and “private” (distanced from social aspects of life) mental imagery is being increasingly shown to play a role in mental health therapy and has been reported to be the most likely used and most effective personal relaxation method (65). Naturalistic Imagery has been shown to reduce stress, shift the autonomic state toward the parasympathetic (66), and attenuate ruminative, self-referential cognitive activity (67–69). Given these insights, externally derived perceptions should have more powerful effects.

Biofeedback is a process that allows for the control of otherwise non-conscious, autonomic functions by providing information on the state of physiological variables (70). It has been successfully utilized in a therapeutic capacity for depression (71, 72), anxiety (73, 74), and insomnia (75). More recently, VR has been integrated with biofeedback with promising results (76, 77). VR provides a more immersive, realistic, comforting, captivating, and multisensory biofeedback experience and thus a keener awareness of the variables targeted for change (70, 78). It also creates a means for social situations and shared networked goals with feedback potential (78). Biofeedback with VR has shown more effective than without VR (79), likely because it reduces hindrances of traditional biofeedback such as lack of meaning to feedback parameters, focus, and motivation, all of which potentially lead to frustration and negative experiences (80, 81).

## A 3D Virtual Space

The cognitive and neural nature of the mind has been pondered by scientists and philosophers for a millennium, but still remains beyond complete understanding. Many modern neuroscientists and philosophers of mind have described the most fundamental cognitive and phenomenal aspect of mind as a virtual three-dimensional (3D) space. This subconscious, 3D, spatial coordinate matrix is proposed to unify all conscious activity and qualia together as they are virtually embedded within its space (82–87). Everything we can sense, think, and feel is experienced within this neurophysiologically generated, virtual space. Even our own experience of self is embedded within it and is centered at its mathematical origin (85). This space forms the bridge This space has been proposed to be formed by fundamental neural oscillations upon which higher frequency banded structures form (88), thus providing an oscillatory foundation for the global bioelectric architecture of the brain (89). Having a spatial structure is considered a requirement of any phenomenal experience to exist in the universe (90). This space may be the key in bridging the gap in understanding on the connection between non-conscious biology and phenomenal consciousness (83). This perspective on mind has not been formally proven and should be an important direction for future research.

## Applications to Virtual Reality Therapy

**Figure 2:**
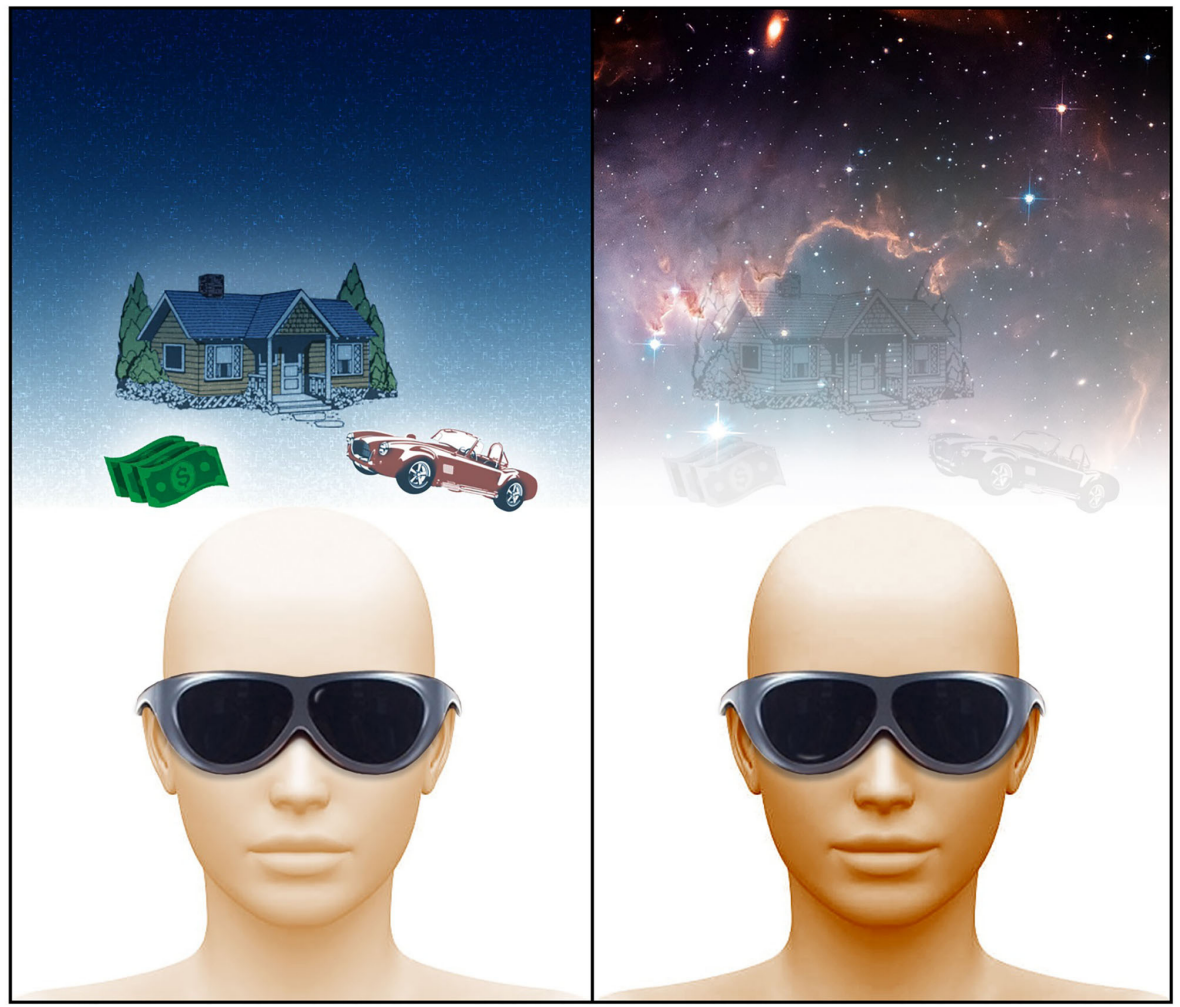
An application of virtual reality therapy using massive expanses of space. In the image on the left, the virtual reality user is not exposed to some sort of massive expanse of space in their virtual or real life experience, and instead is focused on spatially adjacent aspects of their environment. This leads them to continue to dwell on stressful problems that plague them in their spatially adjacent life. With a virtual reality environment that utilizes such a space as shown in the image to the right, the problems which exist on earth (spatially adjacent) fade away as the foundation of mind shifts toward a radically different spatial formation. A virtual reality device concept is shown which is not entirely relevant to this discussion, however, future device concepts of this nature must be comfortable to wear while sleeping for insomnia purposes. Image by Michael Jensen.

Most research has analyzed VR in regards to exposure therapy. In this article however, we discuss potential effectiveness of VR applications centering on use of perceived, massively expansive virtual spaces simulating the expanses of space experienced for instance when looking into the night sky. We suggest the future of mind-body therapy in part lies in virtual reality applications which center around “cosmic relaxation” or other formats which utilize the perception of massive expanses of space. Figure 2 illustrates such an application in use on an example device which is comfortable enough to be worn during sleep. As discussed, there is great potential for breathing and biofeedback techniques for disrupting the vicious cycles. Additional components of such an application could thus include biofeedback-integrated guided exercises for breathing or meditation. Similar to applications such as Apple's “breathe” and the virtual environment with radiating rings representing respiration implemented in (70), artistic and visually captivating animations can be designed into the virtual cosmic expanse which guides the user on these exercises. For instances, the cosmic graphics could take on a breathing motion themselves and colors or more intricate visuals could be used to give biofeedback indicating when the user is matching the breathing pattern well.

The potential effectiveness of this form of virtual reality therapy is necessitated by the perception of such a massive expanse of space. It is ultimately the transformative expansion of the foundation of mind (spatial coordinate matrix) into a radically different state that disassociates one from the problems which plague them in the world founded in the spatially adjacent (compared to such massive expanses of space mentioned) “reality.” We propose that when the mind shifts to this alternate foundation, the architectonics of the brain are shifted as well and the harmful priors and obsessive, ruminative thoughts that characterize the vicious cycles discussed disintegrate. They are simply not an aspect of this new spatial foundation and therefore neural architecture in which the vicious cycles are situated. Once the virtual experience ends, the “spatially-adjacent” neural architecture may return with the same priors and thought patterns, however, breaking the vicious cycles even temporarily should be the key first step, or therapy to repeat, in whittling them down all together. Because these cycles can lead to conditions that are independent of their origin (22), breaking the cycle may be the key to eliminate the conditions.

The disturbance of hyper-arousal and sympathetic hypertonicity with this therapy may also be a crucial factor in breaking such cycles as this mind-body activity likely plays a key role in maintaining the cycles. Many of the problems that trap people into such cycles are socially based. Such virtual environments should be “private” and impersonal enough to disassociate one from their personal life. Because our self-perceptions and personal lives are almost entirely contained in adjacent space, experiencing such massive spaces should have a self-transcendent effect, which is associated with positive mental health outcomes (91, 92). It is this “transcendence” beyond the priors and problems of modern life that has a therapeutic quality, as the cycles and hyper-arousal is often incessant. Such an experience may act as a “pivotal mental state” (a hyper-plastic state fostering rapid and deep learning) which has been argued to mediate psychological transformation and relax negative priors (39). Breaking incessant, negative bodily and mental activity should prevent this negative activity from feeding further activity. The ability for massive spaces to break these cycles is likely why people desire to spend time at the beach or on mountain tops; it shifts their foundation of mind and dissociates them from their stressors.

The technical aspects of such virtual technology this are outside of this article, but further development will need to be done to produce effective technology in creating this experience with devices that are also comfortable to wear in the case of insomnia. The capability to actually create virtually perceptions of such massive spaces may be out of reach for many years, and so we hope to inspire this as an area of technological investigation. We also hope to inspire future research into how different spatial perceptions at great scales affect neural activity, how this relates to vicious cycles, and how this understanding can be used for therapy. Virtual experiences may take the place of therapeutic real-world experiences of massive spaces not easily accessible by most people such as mountain tops, clear night skies, and vast expanses of ocean.

## Conclusion

The emergence of chronic stress due to the onset of depression, anxiety, insomnia and other stress related disorders due to modern life often exacerbates these conditions further leading to a vicious cycle of stress and disorder which is very difficult for many to escape from. We have explored a perspective on a foundation of mind and how this understanding can be utilized to treat the vicious cycles mentioned with virtual reality therapy. We propose that given this nature of mind, virtual reality applications which create the perception of vast expanses of space with for instance cosmic imagery will be effective in breaking people away from the vicious cycles which are ultimately grounded in spatially-adjacent problems. The key to the effectiveness of this form of virtual reality therapy is the perception of vast expanses of space. The technological capability in producing such a virtual experience is currently limited; however, if our perspective holds, then it is a worthwhile development pursuit. This form of application would also benefit from the integration of biofeedback into animated aspects of the virtual environment to enhance the therapeutic quality via breathing and other mind-body techniques. Limitations to exploration of this perspective are largely technological; however, research on the effects of massive space perception should be conducted with real world experiences.

## Data Availability Statement

The original contributions presented in the study are included in the article/supplementary material, further inquiries can be directed to the corresponding author/s.

## Author Contributions

Founding ideas provided by RJ. Article written and background research done by CB along with some idea contribution. Both authors contributed to the article and approved the submitted version.

## Conflict of Interest

The authors declare that the research was conducted in the absence of any commercial or financial relationships that could be construed as a potential conflict of interest.
